# The GYMSSA trial: a prospective randomized trial comparing gastrectomy, metastasectomy plus systemic therapy versus systemic therapy alone

**DOI:** 10.1186/1745-6215-10-121

**Published:** 2009-12-23

**Authors:** Sid P Kerkar, Clinton D Kemp, Austin Duffy, Udai S Kammula, David S Schrump, King F Kwong, Martha Quezado, Barry R Goldspiel, Aradhana Venkatesan, Ann Berger, Melissa Walker, Mary Ann Toomey, Seth M Steinberg, Guiseppe Giaccone, Steven A Rosenberg, Itzhak Avital

**Affiliations:** 1 Surgery Branch, CCR, NCI, Bethesda, MD, USA; 2 Medical Oncology Branch, CCR, NCI, Bethesda, MD, USA; 3 Laboratory of Pathology, CCR, NCI, Bethesda, MD, USA; 4 NIH Clinical Center Pharmacy, Bethesda, MD, USA; 5 Diagnostic Radiology Department, NIH Clinical Center, Bethesda, MD, USA; 6 Pain and Palliative Care Services, NIH Clinical Center, Bethesda, MD, USA; 7 Biostatistics and Data Management Section, CCR, NCI, Bethesda, MD, USA

## Abstract

**Background:**

The standard of care for metastatic gastric cancer (MGC) is systemic chemotherapy which leads to a median survival of 6-15 months. Survival beyond 3 years is rare. For selected groups of patients with limited MGC, retrospective studies have shown improved overall survival following gastrectomy and metastasectomies including peritoneal stripping with continuous hyperthermic peritoneal perfusion (CHPP), liver resection, and pulmonary resection. Median survival after liver resection for MGC is up to 34 months, with a five year survival rate of 24.5%. Similarly, reported median survival after pulmonary resection of MGC is 21 months with long term survival of greater than 5 years a possibility. Several case reports and small studies have documented evidence of long-term survival in select individuals who undergo CHPP for MGC.

**Design:**

The GYMSSA trial is a prospective randomized trial for patients with MGC. It is designed to compare two therapeutic approaches: gastrectomy with metastasectomy plus systemic chemotherapy (GYMS) versus systemic chemotherapy alone (SA). Systemic therapy will be composed of the FOLFOXIRI regimen. The aim of the study is to evaluate overall survival and potential selection criteria to determine those patients who may benefit from surgery plus systemic therapy. The study will be conducted by the Surgery Branch at the National Cancer Institute (NCI), National Institutes of Health (NIH) in Bethesda, Maryland. Surgeries and followup will be done at the NCI, and chemotherapy will be given by either the local oncologist or the medical oncology branch at NCI.

**Trial Registration:**

ClinicalTrials.gov ID. NCT00941655

## Background

Gastric cancer is the fourth most common cancer world-wide. Approximately, 930,000 people are diagnosed each year and the annual mortality is close to 700,000 [1]. In the United States, this year, approximately, 21,500 people will develop gastric cancer and an estimated 10,880 individuals will die from the disease [2]. At the time of diagnosis, 35% of patients have evidence of distant metastases including 31% with peritoneal disease, 14% with metastatic liver disease, and 16% with lung metastases [3-5]. Palliative chemotherapy has been widely used as the treatment of choice for MGC, but only minimal improvements have been observed. Historical data show that patients with MGC who received no treatment had median survival of 3 months, while patients who received modern regimens had median survivals of 7-15 months, and a 2% 5-year survival [6-10].

One of the newer and more promising chemotherapy treatments for MGC is the modified FOLFOXIRI regimen consisting of 5-Fluorouracil, leucovorin, oxaliplatin and irinotecan [11,12]. Results from two phase II trials using the FOLFOXIRI regimen were among the best ever reported for gastric cancer, with overall response rates of 63-66% (CR 2-4% and PR 60-65%), median survival 12-15 months, and median time to progression 7-10 months. The most common grade 3/4 toxicities were neutropenia (12-49% for all cycles), emesis (8-42% for all cycles), grade 2 neuropathy (10%), grade 3 diarrhea (10%), and stomatitis (4%). No grade 4 non-hematologic toxicities were observed.

In regard to locoregional treatments, four separate small studies concluded that complete removal of both the gastric primary and all visible peritoneal disease combined with intraperitoneal chemotherapy (CHPP) was associated with improved survival [13-16]. In the report by Hirose et al., patients who received a CHPP for peritoneal recurrences had improved median survival from 6 to 11 months [15].

Surgical resection of metastatic disease for gastric cancer is not widely performed. We recently performed a critical review of the literature (Kerkar et al., in preparation) and identified 19 studies reporting survival rates on a total of 358 patients who underwent liver resections for MGC. The median of all individual median survivals among reporting studies was 18 months and median five year actuarial survival was 24.5% (range: 0-60%). Although follow up was limited in several studies, 13.4% of patients were still alive at 5 years. In regard to pulmonary metastases from gastric cancer, 22 publications have reported on 45 patients who underwent 49 resections of pulmonary MGC following curative resections of primary gastric malignancies, including patients who have undergone repeat pulmonary resections as well as combinations of hepatic and pulmonary resections (Kemp et al, in preparation). Median overall survival was 21 months [17,18] with multiple reports of survival of greater than 5 years and one study reporting a long term survivor alive 7 years after metastasectomy [19-22].

In light of this retrospective data suggesting that in highly selected patients with limited MGC an aggressive surgical approach plus systemic therapy might improve outcomes, we designed the GYMSSA trial. It is designed to answer the following questions: Is there a group of patients that might benefit from this aggressive surgical plus systemic therapy approach, and if so, how do we choose these patients while avoiding those who will not benefit?

## Methods

### Ethical Approval

The GYMSSA trial was approved by the Institutional Review Board (IRB) of the National Cancer Institute, National Institutes of Health, Bethesda, Maryland.

### Design

**Figure 1:**
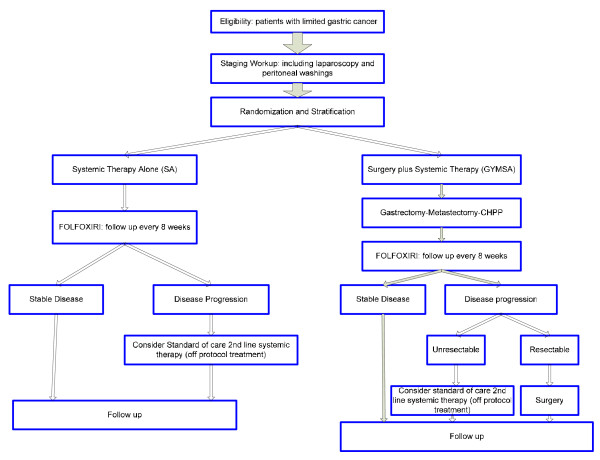
**Trial Schema**.

This is a randomized controlled trial. The trial schema is illustrated in Figure 1. The study will be performed at the Clinical Center of the NIH by the Surgery and Medical Oncology Branches of NCI in Bethesda, Maryland, USA. After thorough staging, including laparoscopy and peritoneal washings, patients will be stratified and randomized to one of two arms: GYMS (gastrectomy, metastasectomy plus systemic therapy) vs. SA (systemic therapy alone.)

### Stratification and Randomization

Registration of patients onto this study will take place within 7 days of obtaining patient consent by faxing a completed eligibility checklist to the Central Registration Office (CRO) at the Clinical Center/NCI. All patients will undergo diagnostic laparoscopy with peritoneal washings to evaluate for occult peritoneal disease and then patients will be stratified and subsequently randomized by the CRO to GYMS or SA arm. The CRO will notify the Study Coordinator of the results of randomization.

Stratification will be done according to the following factors: site of metastases (liver, lung, and peritoneal disease, with positive peritoneal washings stratified as peritoneal disease), time to development of first metastases (less than 1 year versus greater than 1 year), and previous systemic chemotherapy (specifically given for known gastric metastases versus standard adjuvant systemic chemotherapy).

Within 14 days of randomization, patients in the SA arm will begin chemotherapy treatment with FOLFOXIRI (5-FU, leucovorin, oxaliplatin and irinotecan) and those in the GYMSA arm will undergo resection followed by systemic FOLFOXIRI chemotherapy 6-12 weeks after surgery.

As many of the patients referred to this trial will likely have seen 1^st ^line regimens for metastatic disease, we noted that oxaliplatin and/or irinotecan were equally effective in phase-III trials [23] when compared to other common regimens for gastric cancer. Therefore, patients will receive the FOLFOXIRI as described by Masi et. al. from the GONO group [24]. Based on promising results from recent trials and evidence of an acceptable toxicity profile compared to the more standard ECF (epirubicin, cisplatin, and 5-Fluorouracil), we thought that FOLFOXIRI, while unusual, might result in improved survivals. We also felt this line of thought was particularly reasonable since a majority of our patients will have already seen ECF.

### Statistics

The primary objective of the trial is to determine if there is a difference in overall survival among patients with limited gastric cancer metastases who are randomized to receive systemic therapy alone or gastrectomy, metastasectomy plus systemic therapy.

Based upon published data, patients who would be eligible for this trial and who receive systemic chemotherapy alone would be expected to have an estimated 12 month median overall survival from the date of randomization. The primary endpoint of this study will be to determine if the use of gastrectomy, metastasectomy and systemic chemotherapy will result in an 8 month increase in overall survival, to a median of 20 months. Patients will be randomized between systemic therapy alone or gastrectomy, metastasectomy and systemic chemotherapy and followed for overall survival. Stratification will adjust for site of metastases (liver, peritoneal and lung), disease-free interval (DFI), and previous systemic therapy for gastric metastases. Note: positive peritoneal washings will be stratified as peritoneal disease. Kaplan-Meier curves and a two-tailed log-rank test will be the primary analysis methods. Assuming exponential overall survival curves, the hazard rate for the systemic therapy is 0.0578, or approximately a 5.8% probability of death each month when the median survival is 12 months. If we assume that the surgery plus systemic therapy arm has a median overall survival of 20 months, this corresponds to a hazard rate of 0.0347, and the resulting hazard ratio for the comparison of the two overall survival curves would be 1.67. To compare these curves and detect a difference with a 0.05 two-tailed log-rank test, a total of 68 evaluable subjects per arm (136 total) will need to be enrolled over a six year period and followed for an additional two years from the date of entry of the last patient, with 121 total deaths, in order to have 80% power to compare the curves.

Progression free survival will also be evaluated using Kaplan-Meier curves, and a two-tailed log rank test, as a secondary endpoint. In addition, a prognostic factor evaluation using Cox proportional hazards modeling will take place after the study has concluded in order to identify factors that are associated with survival or progression free survival in patients randomized to treatments on this trial; this will also be interpreted as a secondary endpoint.

Quality of life parameters will be measured using evaluations designed for patients with gastric cancer (FACT-Ga, EORTC QLQ-STO22, SROTC QLQ-C30) [25-28] and will be compared using the Wilcoxon rank-sum method between the two study arms at multiple individual time points to determine the differences between the two arms as well as a change from baseline. This evaluation will be considered secondary, and p-values resulting from the analysis will be presented without adjustment for multiple comparisons, but in the context of the number of tests evaluated.

It is expected that 24 patients per year can be accrued onto this trial, and thus accrual will be completed in approximately 6 years. Allowing for a very small number of inevaluable patients, the accrual ceiling will be set at 140 patients.

### Data Monitoring

The study will be monitored by the NCI Center for Cancer Research Data Safety and Monitoring Board (DSMB) on an annual basis to evaluate the safety of the two arms. All adverse events will be recorded. For toxicities possibly attributed to the therapy provided on that arm, serious adverse events (grade 3 toxicities or greater) will be reported according to type of toxicity and maximal grade noted per patient. Comparisons will be made between the two arms using Cochran-Armitage tests for trend, or other appropriate methods, to determine if there is increased toxicity associated with either arm.

In addition, at the first DSMB meeting held following the point at which half of the required total subjects have been enrolled (68 total), a single evaluation for futility will be undertaken. Evaluations for better than expected efficacy will also be made annually, beginning after half the subjects have been enrolled.

### Inclusion and Exclusion Criteria

#### Inclusion Criteria

• Histologically or cytologically confirmed gastric adenocarcinoma with or without prior resection of the primary or metastatic disease

• Limited metastatic disease that is measurable by CT, mandatory PET scan and/or MRI:

- Esophageal invasion <4 cm that does not require thoracotomy (Seiwert II and III lesions)

- Hepatic metastases (unilateral or bilateral, ≤ 5 lesions, ≤ 15 cm total diameter)

- Primary peritoneal metastases (small disease load, ≤ P2 disease) without clinically significant ascites or intestinal obstruction

- Lung metastases (≤ 3 unilateral/bilateral, 9 cm total diameter)

- Patients who present with both hepatic and peritoneal metastases must have no evidence of extensive para-aortic/retro-pancreatic lymph node metastases

• All disease should be deemed resectable to negative margins (NED) based on imaging studies

• Patients with or without prior chemotherapy treatments for metastatic disease will be eligible

• Age greater than 18 years

• Clinical performance status of ECOG ≤ 2

• Life expectancy of greater than three months

• No history of prior/other malignancies within the 2 years prior to enrollment with the exception of basal cell carcinoma

• Laboratory parameters within acceptable limits to undergo surgical resection and/or systemic chemotherapy

#### Exclusion Criteria

• Prior treatment with FOLFOXIRI (treatment with any of the components as separate regimens is allowable)

• Patients with both pulmonary and peritoneal metastases

• Active systemic infections, coagulation disorders or other major medical illnesses (cardiovascular, respiratory or immune system, myocardial infarction, cardiac arrhythmias, obstructive or restrictive pulmonary disease)

• Brain metastases or a history of brain metastases

• Childs B or C cirrhosis or with evidence of severe portal hypertension by history, endoscopy, or radiologic studies

• Weight less than 40 kg

• Significant ascites, greater than 1000 cc in the absence of peritoneal disease

• History of congestive heart failure and/or an LVEF < 40%

• Significant COPD or other chronic pulmonary restrictive disease with PFT's indicating an FEV1 less than 50% or a DLCO less than 40% predicted for age

• Concomitant medical problems that would place the patient at an unacceptable risk for a major surgical procedure

### Intervention

#### GYMS Arm

All patients randomized to the GYMS arm will undergo surgical resection of their primary and/or metastatic tumors within 14 days of randomization. Patients will undergo surgery only at the Surgery Branch, NCI with the goal of resection of all visible metastases to leave patients with no evidence of disease.

Patients with proximal gastric lesions will undergo total gastrectomy with a 2-4 cm esophageal margin, those with cardiac or fundic tumors with undergo total gastrectomy, and those with distal lesions will undergo a radical subtotal gastrectomy. All procedures will include a modified D2 lymphadenectomy and a Roux-en-Y reconstruction.

Pulmonary and hepatic metastasectomies will be performed in a standard fashion at the discretion of the attending thoracic or surgical oncologist. Patients who have peritoneal disease will undergo complete peritonectomy and CHPP with intravenous 5-FU/leucovorin and intraperitoneal oxaliplatin. Patients who present with intrabdominal disease without peritoneal involvement will undergo a limited peritonectomy followed by CHPP with intravenous 5-FU/leucovorin and intraperitoneal oxaliplatin.

Patients who present with synchronous intrabdominal metastatic disease (hepatic or peritoneal) at time of initial diagnosis will undergo a gastrectomy and appropriate metastasectomy. For patients with synchronous pulmonary metastases, a decision will be made by the thoracic and surgical oncologist as to the optimal timing of gastrectomy and metastasectomy (synchronous or staged resections) with a delay of no more than 8 weeks between procedures.

All patients must begin systemic FOLFOXIRI chemotherapy within 8 weeks after surgical resection.

#### SA Arm

Patients randomized to the systemic therapy arm (SA) will receive systemic FOLFOXIRI chemotherapy either by their home medical oncologist or by the Medical Oncology Branch, NCI. Only palliative surgery will be allowed as per standard of care for issues such as obstruction, bleeding and/or intractable pain. No crossover will be allowed.

### Data Collection and Evaluation

Data will be collected using the NCI C3D web based data collection system.

Baseline history and physical examinations, laboratory studies, radiological imaging, and quality of life questionnaires will be obtained for patients on both arms. Additional testing will be performed prior to treatment as dictated by patient characteristics.

Following randomization, followup will be every 2 months during chemotherapy, and then every 3 months for the first 2 years, every 6 months for the subsequent 3 years, and yearly thereafter. Follow-up will consist of interval history and physical examination, laboratory studies, radiological imaging, and quality of life questionnaires.

Lesions will be evaluated using the RECIST criteria. The duration of overall response will be measured from the time measurement criteria are met for partial response (PR) or complete response (CR), until the first date that recurrent or progressive disease is objectively documented (taking as reference for progressive disease the smallest measurements recorded since the treatment started).

The duration of overall complete response will be measured from the time that measurement criteria are first met for CR until the first date that recurrent disease is objectively documented. This study will utilize common terminology criteria for toxicity and adverse event reporting (version 4.0; http://ctep.info.nih.gov).

### Safety and Monitoring Plan

Careful evaluation to ascertain the toxicity and clinical response will be performed. The principal investigator will monitor the data and toxicities in order to identify trends quarterly. The Principal Investigator will be responsible for revising the protocol as needed to maintain safety. The NCI IRB will review submitted adverse events monthly to also evaluate trends and will require a follow up plan from the Principal Investigator whenever a trend is identified.

The trial will be monitored at least annually by the NCI DSMB. Interim outcome results will not be revealed to the investigators of the trial; results will be presented to the investigators prior to final accrual only if the DSMB recommends early termination of the trial. Until 68 patients have been randomized, only toxicity and adverse events will be examined at each review. Once 68 patients have been randomized, interim evaluations will be performed to determine whether there is sufficient evidence to terminate accrual because of a better than expected improvement in overall survival. At the first such review of outcomes, a single evaluation for futility will also be performed.

### Informed Consent

All patients are thoroughly screened prior to initial consultation at the NIH. During the initial consultation, the patient, along with family members, is presented a forthright and detailed overview of the treatment option available to them at the NIH. The experimental nature of the treatment, its theoretical advantages and disadvantages, and an overview of the operative procedure and anticipated convalescence are presented. The fact that the patient may undergo an operative procedure in order to receive therapy without any assurance of benefit, the aggressive nature of the treatment, and the likelihood of serious or potentially life-threatening complications are presented. The Informed Consent document is given to the patient and they are asked to review it, make notes, and follow-up with a phone call to the physician or nurse investigator in order to have any additional questions answered prior to considering treatment on protocol. The research nurse, Principal Investigator, or designee is responsible for obtaining consent from the patient upon admission. The signed consent will be verified by the physician responsible for the care of the patient. Patients are free to withdraw from the study at any time without any obligation.

### Endpoints/Follow-up of Study

#### Primary Objective

• Determine whether a therapeutic approach that includes gastrectomy and/or metastasectomy plus systemic therapy is superior to the standard of care approach that includes systemic therapy alone in terms of over-all survival for patients who present with limited metastatic gastric cancer.

#### Secondary Objectives

• Compare progression free survival between the two study arms

• Analyze prognostic factors and generate potential selection criteria for patients who present with limited gastric cancer metastases that might benefit from gastrectomy and/or metastasectomy plus systemic therapy.

• Determine if surgical resection impacts the dosing and duration of subsequent chemotherapy administration

• Compare quality of life (QOL) parameters between the two study groups. We will use tools specifically developed for assessment of QOL in gastric cancer patients: FACT-Ga, EORTC QLQ-STO22 and the SROTC QLQ-C30 [25-28].

• Determine and compare patterns of disease recurrence between the two therapeutic approaches and their clinical implications.

## Discussion

Overall, gastric cancer is the 14^th ^most common cancer in the United States, and accounts for 1.5% of all new diagnoses and 5.2% of all cancer deaths [29]. Long-term survival for patients with metastatic gastric cancer is dismal, and any improvement in overall survival will be a significant advance. Over the past several decades, improvements in the understanding of anatomy, physiology, perioperative care, and surgical technique have reduced operative morbidity and mortality for major resections such as hepatectomies, thoracotomies, and peritoneal stripping to less than 4% [30]. Additionally, the latest trials utilizing the FOLFOXIRI chemotherapeutic regimen have demonstrated improvements in survival for patients with MGC without any additional major grade 4 non-hematologic toxicities [11,12].

The optimal treatment for patients with MGC is not known. The benefit of gastrectomy and metastasectomy plus systemic chemotherapy versus systemic chemotherapy alone has not been studied. This trial will either help validate or reject the notion based on retrospective data that aggressive surgical resections in combination with systemic chemotherapy might improve outcomes in metastatic gastric cancer.

## Competing interests

There are no declared competing interests. There is no financial support by private institutions or companies.

## Authors' contributions

IA is the Principal Investigator for the study described in the manuscript. IA, SK, and CK made significant contributions to the protocol validity, design and drafting/revising the manuscript. SR revised the manuscript and gave approval for the final version to be published. All authors participate in the study by enrolling patients and collecting data. SS developed the statistical considerations for the trial. SK, CK, AD, UK, DS, KK, MQ, BG, AV, AB, MW, MT, GG, SR, and IA contributed to the scientific accuracy of the manuscript.
